# Long-Term Potato Virus X (PVX)-Based Transient Expression of Recombinant GFP Protein in *Nicotiana benthamiana* Culture In Vitro

**DOI:** 10.3390/plants10102187

**Published:** 2021-10-15

**Authors:** Yana Sindarovska, Mykola Kuchuk

**Affiliations:** Department of Genetic Engineering, Institute of Cell Biology and Genetic Engineering of NAS of Ukraine, 148 Akad. D.K. Zabolotnogo Str., 03143 Kyiv, Ukraine; nkuchuk@icbge.org.ua

**Keywords:** recombinant proteins, transient expression, *Nicotiana benthamiana*, potato virus X (PVX), viral vector, plant tissue culture, green fluorescent protein (GFP)

## Abstract

**Simple Summary:**

Nowadays, the global recombinant protein market is valued at USD 125 billion. While the main producers of recombinant proteins are bacterial and mammalian cell cultures, plants have been intensively explored as an alternative platform for obtaining recombinant proteins (biopharming). Plants are safer systems, as they are free of human pathogens, oncogenic sequences, or bacterial endotoxins. Here, we proposed a new system for the production of recombinant proteins through transient expression technology in *Nicotiana benthamiana* plants grown in aseptic conditions (in vitro). Closed in vitro systems (cell tissue cultures) are well controlled; thus, they are preferable for recombinant protein production, as they can be more easily standardized for the pharmaceutical market purposes, and transient expression allows high levels of the recombinant proteins to be produced. We used an agrobacteria-delivered vector containing phytopathogenic virus (potato virus X) sequences to create plant tissue culture with prolongated transient expression of recombinant reporter green fluorescent protein (GFP). The mean of GFP was 18% of the total soluble cell proteins (TSP) (0.52 mg/g of fresh leaf weight (FW), and the best result reached 47% TSP (2 mg/g FW). The system can be a new technique for biopharming, combining the advantages of transient expression and cell tissue cultures.

**Abstract:**

Plant molecular farming has a great potential to produce valuable proteins. Transient expression technology provides high yields of recombinant proteins in greenhouse-grown plants, but every plant must be artificially agroinfiltrated, and open greenhouse systems are less controlled. Here, we propose to propagate agrobacteria-free plants with high-efficient long-term self-replicated transient gene expression in a well-controlled closed in vitro system. *Nicotiana benthamiana* plant tissue culture in vitro, with transient expression of recombinant GFP, was obtained through shoot induction from leaf explants infected by a PVX-based vector. The transient expression occurs in new tissues and regenerants due to the natural systemic distribution of viral RNA carrying the target gene. Gene silencing was delayed in plants grown in vitro, and GFP was detected in plants for five to six months. Agrobacteria-free, GFP-expressing plants can be micropropagated in vitro (avoiding an agroinfiltration step), “rejuvenated” through regeneration (maintaining culture for years), or transferred in soil. The mean GFP in the regenerants was 18% of the total soluble proteins (TSP) (0.52 mg/g of fresh leaf weight (FW). The highest value reached 47% TSP (2 mg/g FW). This study proposes a new method for recombinant protein production combining the advantages of transient expression technology and closed cultural systems.

## 1. Introduction

In the last three decades, starting from the first publication announced about successful production of plant-made pharmaceutical [1], plants have been intensively explored as an alternative platform for obtaining recombinant therapeutic proteins. This industry platform is known as plant molecular farming. Nowadays, several companies around the world develop plant-based pharmaceuticals, such as Protalix BioTherapeutics, Medicago, Kentucky Bioprocessing, Greenovation Biotech, Mapp Biopharmaceutical, PlantForm, Icon Genetics, Nomad Bioscience, etc. There are two widely used ways for obtaining recombinant proteins in plants: (i) creating transgenic plants or plant cells with stable transgene expression, which is integrated into the plant genome, or (ii) infection of plant cells with bacterial T-DNA or viral RNA, leading to temporary gene circulation and transient gene expression. Transgenic plants demonstrate the stable protein production and transmission of a target gene and can be scaled in fields or bioreactors, but the levels of recombinant proteins are typically low, and currently, plant-made biopharmaceuticals are rare product on the market. Protalix BioTherapeutics is the first company to have the plant-made product produced by transgenic carrot cell culture and approved for administration by the US Food and Drug Administration. Some other companies (Medicago, Icon Genetics, PlantForm, Nomad Bioscience) use the *Agrobacterium-*mediated transient expression technique to produce biopharmaceuticals in greenhouse-grown *Nicotiana benthamiana* plants. Transient gene expression allows high yields of recombinant proteins (up to 60–80% TSP) [2,3,4] to be obtained in a few days. However, the recombinant protein production is temporary and usually disappears after 1–2 weeks, and unstable greenhouse conditions (e.g., pathogen attack or variable soil nutrient composition) affect the recombinant protein levels. The transient expression occurs without transgene inheritance, so to obtain the product, an artificial infection (e.g., vacuum agroinfiltration) must be repeated with every plant, thus complicating the manufacturing process [5]. Typically, an agroinfiltration buffer includes MES (for pH stabilization) and acetosyringone (for bacterial *vir* genes induction) [6,7], which are expensive compounds for the commercial process and must be replaced by cheaper ones, e.g., distilled water [8], thus reducing transfection efficiency. In commercial facilities, large vacuum vessels (up to 7000 L) are used for plant agroinfiltration (Medicago, [9,10]). Therefore, additional attention must be paid to the deactivation of unutilized transgenic bacteria that may transfer to the environment and lead to soil and water pollution. An alternative spray-based technique alleviates the need to use the vacuum chambers [4,5,11] but does not eliminate the artificial infection itself and increases an environmental pollution hazard caused by agrobacteria.

Viral-based vectors allow high levels of valuable proteins to be obtained after transient gene expression [4,5,12,13,14,15,16,17,18,19]. Currently, viral-based vectors are often incorporated into agrobacterial cells to increase the number of simultaneously infected plant cells during the infiltration process [20]. As the agrobacterial genes ensure the transfer of a target gene to the plant cells, the viral-based expression vectors often lack the viral genes coding movement proteins to reduce the plasmid size.

Potato virus X is a plant pathogenic filamentous potexvirus with plus-sense single-stranded RNA that is mechanically transmitted to the host plants, especially to *Solanaceae* family species [21]. To facilitate cell-to-cell and vascular transport, PVX requires the triple gene block (TGB) movement proteins and the viral coat protein [22]. Vascular transport of the viral RNA provides systemic infection in distal upper leaves. Moreover, TGBp1 protein counteracts the virus-induced gene silencing [21]. PVX is a model object for plant virology [23], which has been studied as a pathogen affecting the quality and yield of potatoes and other crops in commercial production [24,25,26]. Detailed studies have revealed the fundamental mechanisms underlying plant–virus interactions and gene silencing, thus promoting gene functional analysis experiments [27,28] and the development of PVX-based expression vectors for the production of recombinant proteins in plant systems [13,15,21,29,30,31,32,33]. Typically, PVX-based vectors are used for the infection of greenhouse-grown plants. However, it was reported that plant viruses, including PVX, can persist in tissue cultures for years [34,35], thereby suggesting the possibility of using plant viral vectors for transient expression of recombinant proteins in plant tissue cultures.

Here, we propose a new system for the high-yield production of recombinant proteins, which is based on long-term transient gene expression in vitro. This approach combines the advantages of transient expression as the target protein reaches the high levels, and plant tissue cultures are made: (i) the plant cultivation and protein production occur under well-controlled conditions and (ii) plant material (producer of recombinant protein) is maintained for a long time (years) due to natural distal distribution of viral RNA and micropropagation. A PVX-based vector system with genes coding the viral RNA polymerase, the triple gene block proteins, the coat protein, and the reporter protein was used to create tissue culture of *Nicotiana benthamiana* plants expressing GFP via shoot regeneration in vitro. Transient GFP expression in plants growing in vitro was stable enough and was detected without notable gene silencing for up to six months. The average GFP level was about 18% of TSP. A proposed approach can be used as a closed well-controlled in vitro system for recombinant protein production or can be used as an alternative to the existing *Agrobacterium-*mediated transient expression technique with greenhouse-grown plants, as it alleviates the necessity of agroinfiltration step for the recombinant protein production. The regenerated plants growing in vitro can be used for the different purposes: (i) micropropagated to multiply the plants producing the target protein; (ii) transferred on regeneration media for tissue culture “rejuvenation”; (iii) transferred to soil to obtain adult plants producing the recombinant protein.

## 2. Results

### 2.1. In Vitro Tissue Culture Establishment: Regeneration and Micropropagation of Plants with Long-Term Transient Expression of Recombinant Protein

**Figure 1:**
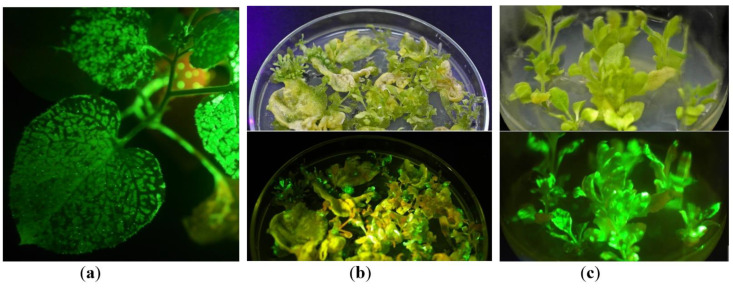
Steps for obtaining in vitro tissue culture transiently expressing the target *gfp* gene: (**a**) plant infected with PVX-based vector via agroinfiltration: GFP production in the upper leaves after systemic virus infection (under UV light); (**b**) the GFP-expressing explants on MSR medium; (**c**) the young GFP-expressing regenerants on MS medium. (**b**,**c**) photos were made under visible (up) and UV (down) light. Green fluorescence (under UV light) indicates GFP accumulation. All photos in UV light were made using an orange filter.

To obtain in vitro tissue culture of *Nicotiana benthamiana* plants with transient expression of the *gfp* gene, a PVX-derived vector system incorporated into *Agrobacterium tumefaciens* cells was used. PVX-based cassette consists of (i) the potexviral RNA-directed RNA polymerase gene providing a high level of RNA replication; (ii) the potexviral coat protein gene and the triple gene block providing systemic distribution of viral RNA in the upper non-inoculated leaves of infected plants; (iii) the *gfp* gene placed under a viral subgenomic promoter. Initially, two plant species—namely, *Nicotiana benthamiana* and *N. excelsior*, with high sensitivity to viruses were tested [3]. Mature leaves of six-to-eight-week-old *N. benthamiana* and *N. excelsior* plants grown in a greenhouse were infected with agrobacterial cells carrying PVX-based plasmid and a plasmid carrying *p19* gene, which coded a suppressor of gene silencing driven by 35S promoter of CaMV. Green fluorescence indicated on GFP accumulation appeared within infiltration areas 3–4 days after infection in *N. benthamiana* leaves and delayed a few days in *N. excelsior* leaves. After 2–5 days, spots/sectors with bright green fluorescence started to emerge on the upper young non-infiltrated leaves of *N. benthamiana,* indicating the long-distance systemic infection and spread of viral RNA carrying *gfp* gene (Figure 1a). The plasmid carrying the *p19* gene lacked movement protein genes, and the produced RNA transcripts did not spread in the upper young non-infiltrated leaves. Systemic infection was less pronounced in *N. excelsior* plants: spots appeared later and were small. Leaves with visible fluorescence (infiltrated and systemic) were cut off, and the surface was sterilized with soap and ethanol (see Materials and Methods Section). The sterilization procedure was simple and effective. Then, leaves were cut on 2–3 explants and placed on the regeneration medium (MSR) [36,37] to induce the formation of shoots with transient gene expression in vitro. After several repeats, it was noticed that leaves (explants) that were initially infiltrated with bacterial suspension always showed extensive bacterial contamination incompatible with the next regeneration procedures. In contrast, systemically infected leaves were successfully transferred in aseptic conditions, mainly without bacterial or fungal contamination. As *N. excelsior* showed weak systemic distribution, and the regeneration procedure was not effective for it, this species was excluded from further investigation. *N. benthamiana* was used for the following experiments. After 2–3 weeks laying on MSR, calli appeared on the explants. Soon thereafter, shoots with very bright green fluorescence, indicated on high GFP content in tissues, started to emerge on calli (Figure 1b). Regeneration was effective with *N. benthamiana* explants, and more than 50 regenerants producing GFP were obtained from 4–5 agroinfiltrated plants (Figure 1c). Typically, 4 to 10 regenerants can be produced by one explant. Almost all obtained regenerants expressed GFP at high levels that were visually assessed; however, few regenerants did not display GFP expression at all, no matter how long they grew in vitro. The regenerants that could be safely detached from callus were transferred to a regular MS medium [38] without additives. MS was used subsequently to maintain the plant tissue culture.

**Figure 2:**
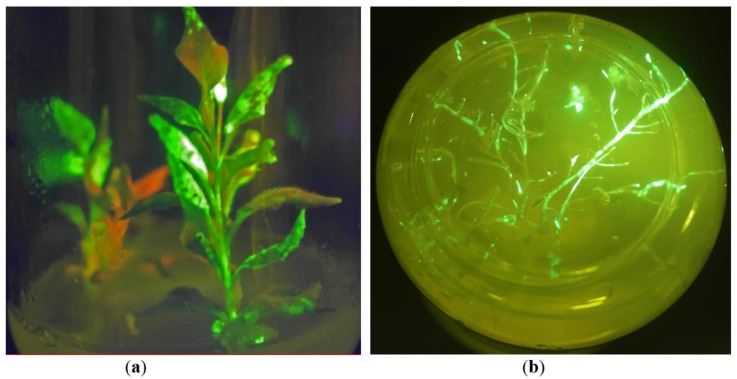
*N. benthamiana* regenerants growing in vitro (under UV light): (**a**) two-month-old regenerants; (**b**) roots of the GFP-expressing regenerants. The green color shows GFP fluorescence, and the red-brown color displays chlorophyll fluorescence; light gleams emerge in the places with very high GFP fluorescence (that correlated with GFP production).

The infection process occurred in the regenerants in the same way as in the wild-type plant samples: firstly, the lower leaves were infected, and then green fluorescence appeared on the upper leaves proving systemic distribution of viral RNAs with the *gfp* gene. Roots started to appear on the regenerants after the next two weeks. When roots appeared, a part of the regenerants was transferred to soil for further greenhouse experiments. Another part of the regenerants was left for plant micropropagation and maintaining tissue culture. Our observations reveal that the regenerants growing in vitro showed a significant delay in growth rate, compared with the plants growing in soil; nevertheless, most of them showed bright green fluorescence for several weeks, both in leaves and in roots (Figure 2a,b). Few regenerants were maintained in vitro conditions for more than six months, and they still demonstrated GFP production in upper and middle leaves during this period. PCR analysis on the *virD1* gene confirmed that the regenerants were free of *Agrobacterium* contamination. As an internal control of DNA isolation, the samples were tested by PCR analysis for the presence of the housekeeping *actin* gene. Electrophoretic separation of PCR products showed the presence of the expected band (Figure S1). PCR analysis confirmed that there was no *gfp* gene integration in genomic DNA of the GFP-expressing regenerants (Figure S2). Moreover, to confirm the transfer of viral RNA in the young uninfected leaves and *gfp* gene expression from the viral RNA (transient expression without transgene integration), a simple virological test was made: sap from the upper systemically infected leaves was filtrated through 0.22 µm membrane filter (to exclude bacterial contamination) and then new plants were infected with throughflow. After two weeks, young leaves showed bright green fluorescence, thus confirming that RNA molecules carrying the target *gfp* gene entered the plant cells and induced GFP expression. Further, leaves of the GFP-expressing regenerants were put on MSR regeneration medium again to “rejuvenate” the tissue culture and to confirm continuity of the recombinant protein production process. New young regenerants were successfully obtained from GFP-expressing plants growing in vitro without agroinfiltration step, and the second generation of the regenerants demonstrated the same high levels of GFP as the first generation of the regenerants. Thus, in vitro tissue culture of *N. benthamiana* plants with long-term transient expression of the *gfp* gene was established.

### 2.2. GFP Production in N. benthamiana Regenerants

**Figure 3:**
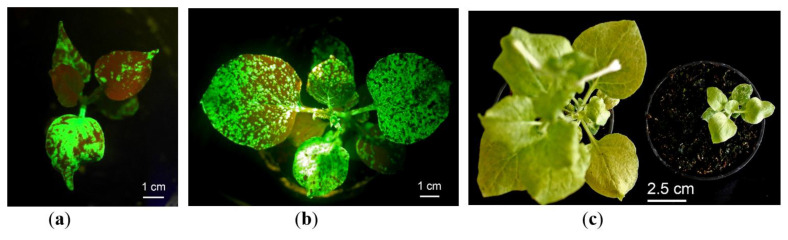
The GFP-expressing regenerants in soil. The regenerants just after transferring to soil (**a**) or growing in greenhouse conditions for two weeks (**b**) under UV light; (**c**) the regenerants of the same age transferred to soil and grew in greenhouse condition for one month; (**a**,**b**) the green color shows GFP fluorescence, and the red-brown color displays chlorophyll fluorescence; light gleams emerge in the places with very high GFP fluorescence (that correlated with GFP production).

**Figure 4:**
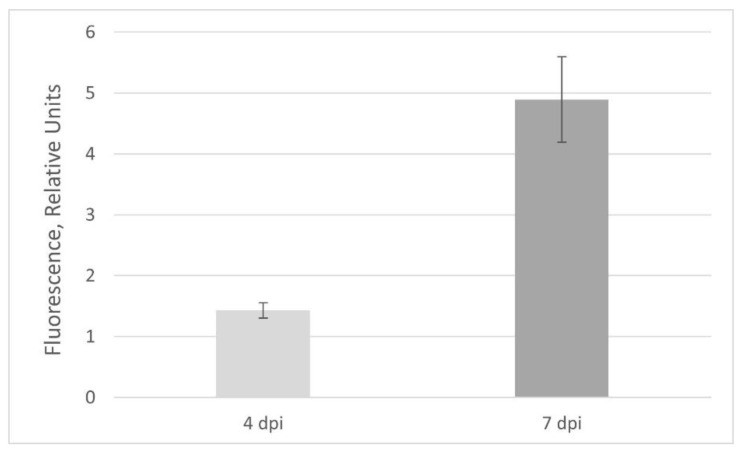
Comparison of the relative GFP fluorescence in the attached leaves of positive control *N. benthamiana* plants agroinfiltrated with the same PVX-based vector depending on the day post infiltration (dpi). Measurements were made at an excitation wavelength of 395 nm and an emission wavelength of 510 nm. Bar means a standard error (SE).

To assess the GFP production in the obtained regenerants, we transferred the plants grown in vitro to soil. Transferring the regenerants from nutrient medium (aseptic in vitro conditions) to soil (greenhouse conditions) often induced extensive plant growth, and after 4–6 weeks, most of the regenerants restored their phenotype and were similar to the normal adult six-week-old *N. benthamiana* plants (Figure 3). However, about 1 in 10 plants still demonstrated dwarfism (Figure 3c). After visual inspection, it was concluded that plant growth rate did not correlate with the GFP production: some of the dwarf regenerants displayed very bright fluorescence, while some of the well-developed regenerants showed weak fluorescence. When plants grew in soil, RNA transcripts infected the new upper leaves but more efficiently the smaller leaves rather than larger ones that were detected visually via green fluorescence. If a vacuum or syringe agroinfiltration method is used, almost all plant resources switch on the target protein production, and the major plant protein RuBisCO, which is involved in inorganic carbon fixation, decreases notably [2,3]; thus, plants cannot live a long time after agroinfiltration. In our experiments, the leaves of positive control *N. benthamiana* plants agroinfiltrated with the same PVX-based vector mixed with a vector carrying *p19* gene started to wither notably and decreased their fluorescence 6–7 days post infiltration (dpi). On the other hand, GFP fluorescence in control plants did not reach the maximum mean after 4 dpi (Figure 4). Thus, recombinant proteins in totally agroinfiltrated plants must be harvested in a short period of time. In contrast, after systemic viral infection, the regenerated plants grew quite normally for a month and enlarged their leaf biomass, and therefore increased the total recombinant protein content.

After 4–6 weeks of growing in soil, the well-developed regenerants were tested for the target protein production. In total, 16 regenerants of different ages (that were grown in vitro for 1–4 months and then grown in soil for 1–1.5 months) were taken in the experiments. The regenerants obtained in the same way as experimental ones but were not infected with a viral vector, or the wild-type plants obtained directly from the seeds were used as the negative controls. Plants infected with the same PVX-based vector via agroinfiltration were used as a positive control. Visual inspection under daylight showed that part of GFP-expressing regenerants had green leaves with light green chlorosis areas, a typical virus disease symptom. These chlorosis areas corresponded to the fluorescence area under UV light and, accordingly, the recombinant protein accumulation, while other regenerants had noticeable yellowing of all foliage after growing in the greenhouse for this period (Figure 3c). Completely yellowed leaves did not express GFP anymore. In contrast, the GFP-expressing regenerants growing in vitro remained green for a long period of time (more than two months). The regenerant R1, which demonstrated the best GFP expression level, also had green leaves (Figure S3). Thus, leaf pigmentation can be an indicator for the best harvesting of the target protein. We also noticed that after one-and-a-half months of growing in soil under greenhouse conditions, the GFP expression in the regenerants started to decrease, and after two months, fluorescence almost completely disappeared because of gene silencing. In contrast, as was mentioned above, the green fluorescence was detected in vitro regenerants up to six months.

**Figure 5:**
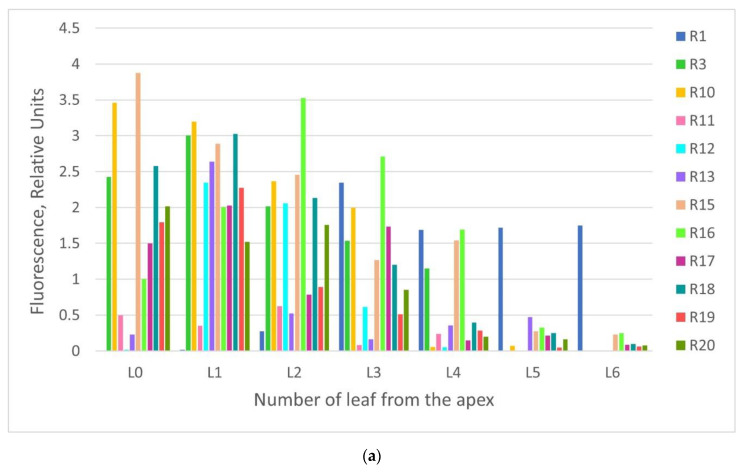

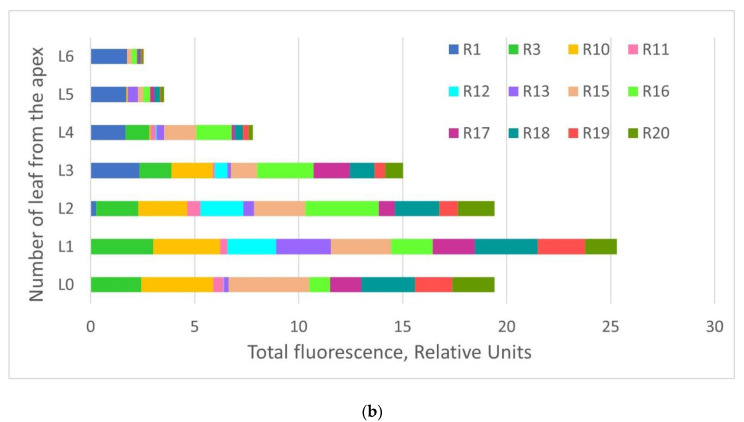
Comparison of the relative GFP fluorescence in the attached leaves of the regenerants depending on leaf order: (**a**) comparison of GFP fluorescence between the individual plants and leaves; (**b**) the combined data of GFP fluorescence in the separate leaves depending on the order from the apex. Measurements were made at an excitation wavelength of 395 nm and an emission wavelength of 510 nm. R1–R20—the tested regenerants grown in the greenhouse: R1–R13—the first generation regenerants (obtained from initially infected explants); R15–R20—the second generation regenerants (obtained from the leaves of the first generation regenerants growing in vitro). L0–L6—position of leaf from the apex: L0—not fully expanded upper leaf; L1—first fully expanded upper leaf, etc.

**Figure 6:**
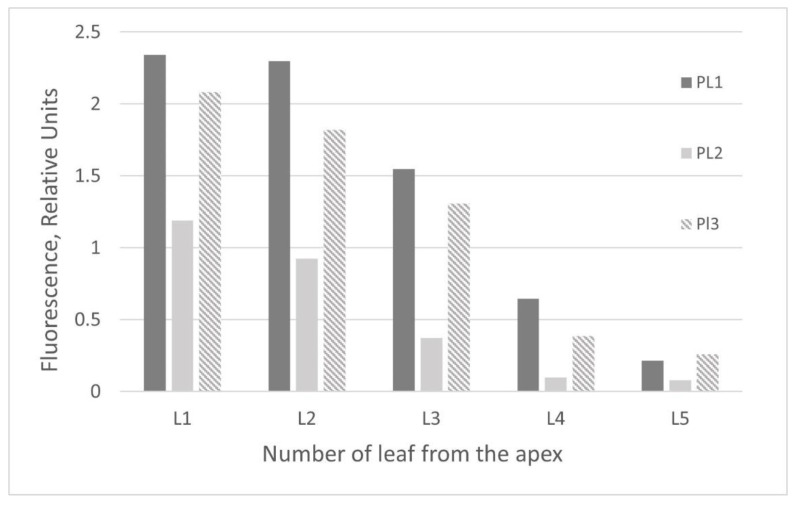
Comparison of the relative GFP fluorescence in the attached leaves of positive control *N. benthamiana* plants agroinfiltrated with the same PVX-based vector depending on leaf order. Measurements were made on the 5th day post infiltration at an excitation wavelength of 395 nm and an emission wavelength of 510 nm. L1–L5—position of leaf from the apex.

The recombinant protein accumulation was assessed in the intact attached leaves by spectrofluorimetric measurements of GFP fluorescence (measured in the relative units). Then, it was assessed by spectrofluorimetric measurements in the total soluble protein extracts prepared from whole GFP-expressing foliage of the individual regenerants (measured both in the relative units and in the absolute values). Spectrofluorimetric data revealed that GFP levels varied greatly among the individual leaves on the same plant and between the plants (Figure 5a). However, some predictions regarding protein production in the separate leaves can be made based on shown data (Figure 5b). High yields of the recombinant protein were obtained in the five upper leaves, starting from leaf “zero” (L0) (not fully expanded upper leaf) from the apex. Similar results (there was a variety of GFP expression between the individual plants, and between the separate leaves depending on leaf order) were observed for the control plants agroinfiltrated with the same PVX-based vector (Figure 6).

**Figure 7:**
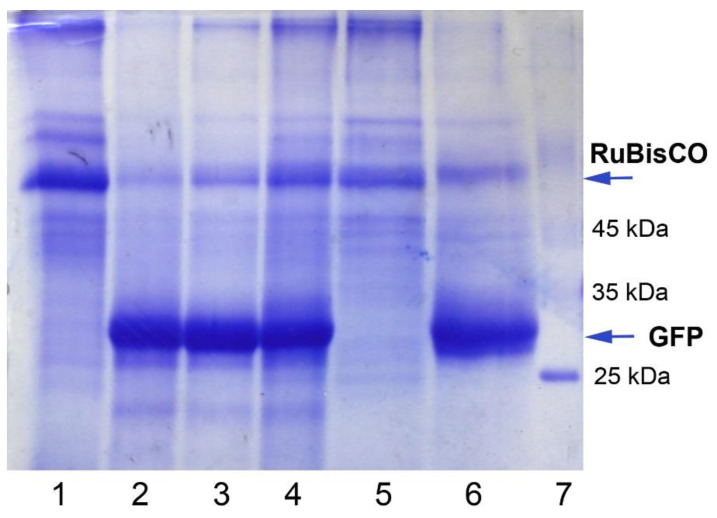
Electrophoretic separation of the protein extracts in 12% SDS–PAGE after staining with Coomassie Brilliant Blue (the equal protein amounts were added in the wells): 1—extract from a control *N. benthamiana* plant (wild type); 2—extract from the upper leaf of GFP-expressing *N. benthamiana* regenerant; 3—extract from the leaf of *N. benthamiana* plant infected with PVX-based plasmid via agroinfiltration; 4—extract from the upper leaf of *N. benthamiana* plant systemically infected after agroinfiltration (3 and 4 are the positive control extracts); 5—extract from a control *N. excelsior* plant (wild type); 6—extract from the leaf of *N. excelsior* plant infected with PVX-based plasmid via agroinfiltration; 7—a protein marker ladder. The arrows indicate a large subunit of RuBisCO (the most abundant plant protein) and recombinant GFP protein accumulation.

To evaluate the highest levels of GFP that can be produced by the regenerants, the upper leaves with very bright fluorescence were used to prepare the total soluble protein extracts, and native and SDS-reducing PAAG gel electrophoresis (PAGE) were carried out. Visual inspection (under UV light) of the proteins separated by native PAGE proved the presence of recombinant GFP in the prepared extracts with the expected mobility. Recombinant GFP was also detected as the major protein band with corresponding molecular weight (27 kDa) in the experimental samples (extracts prepared from the leaves of GFP-expressing regenerants), and the positive controls (extracts prepared from the leaves infected with PVX-based vector via agroinfiltration, and extracts prepared from the systemically infected leaves after agroinfiltration) after reducing SDS–PAGE (Figure 7). In contrast, the level of a large subunit of RuBisCO, a dominant plant cell protein, significantly decreased, indicating that plant resources were used for recombinant protein synthesis. GFP levels were comparable for the regenerants and for the agroinfiltrated plants. As it was calculated using ImageJ image analysis software, GFP production in the individual leaves of the regenerants could reach considerable values (up to 50%–80% of the total soluble cell proteins).

As levels of the target protein varied notably between the leaves, and therefore a leaf position can influence the obtained results, all GFP-expressing foliage was harvested to estimate a quantity of the recombinant protein that can be produced by one regenerant. While in this experiment, GFP was extracted only from the leaves, it was also detected in stems and roots. GFP fluorescence in the total protein extracts was measured for 10 regenerants. The relative fluorescence in nine of ten protein extracts was comparable, but the extract of one regenerant (R1) showed a significant difference from others, surpassing them about five times (the emission peak of GFP fluorescence is 510 nm, Figure S4). The negative control regenerant (RC1) demonstrated just background fluorescence.

**Figure 8:**
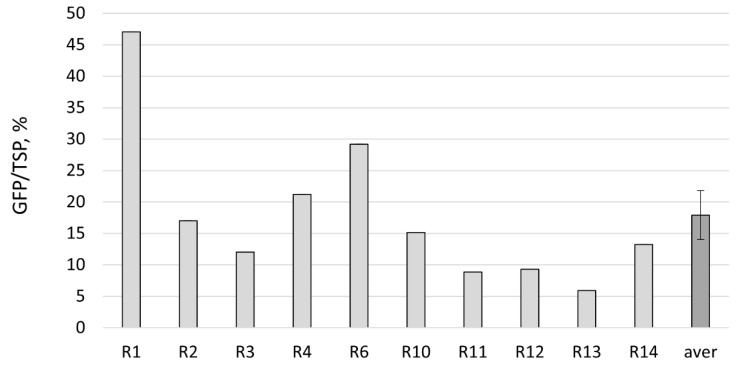
The GFP content in the protein extracts of *N. benthamiana* regenerants prepared from the total GFP-expressing foliage calculated as a percentage of the total soluble cell proteins (TSP): R1–R14—the tested regenerants transferred from in vitro conditions to soil and grown for 1–1.5 months in the greenhouse; arev—the average mean. The regenerants R1 and R3 grew in vitro for one month before transferring to soil; R2, R4, and R6 grew in vitro for two or two-and-a-half months; R10–R14 grew in vitro for three-and-a-half or four months. Bar means a standard error (SE).

**Figure 9:**
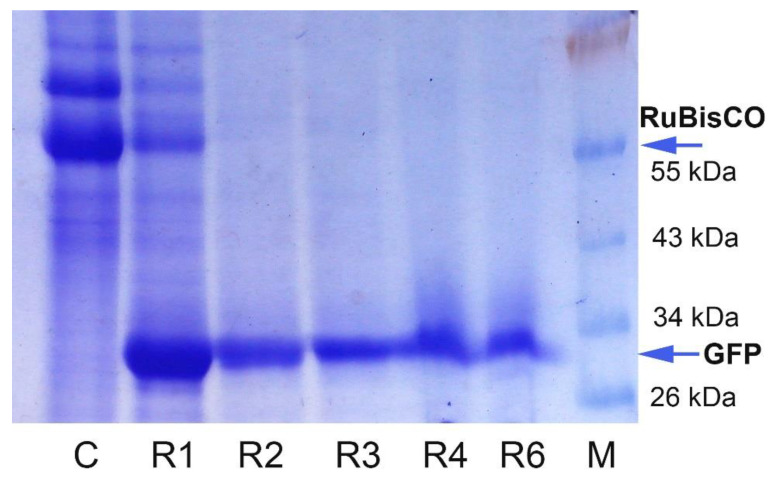
Electrophoretic separation of the protein extracts in 12% SDS–PAGE after staining with Coomassie Brilliant Blue: wells were loaded with the equal volumes (30 µL/well) of crude extracts prepared from the total GFP-expressing foliage of the regenerants (fresh weight leaf material was ground in two volumes of extraction buffer and mixed with 3x loading buffer). C—the protein extract of a control plant (the regenerant without viral RNA); R1–R6—the protein extracts obtained from total GFP-expressing foliage of the regenerants (R1, R2, R3, R4, and R6, respectively); M—a protein marker ladder. The arrows indicate a large subunit of RuBisCO (the most abundant plant protein) and recombinant GFP protein accumulation.

Further, GFP content was calculated in the absolute values using purified standards: as a percentage of the total soluble cell proteins (TSP), as the amount per fresh leaf weight, and as the amount harvested from the individual regenerants. The average percentage of GFP to TSP in the regenerated plants was 17.9 ± 3.9% and reached up to 47% (the variation range was 5.9–47.1%) (Figure 8). The control agroinfiltrated plants collected on 6–7 dpi showed similar results, with 19.8 ± 2.7% (the variation range was 6.7–32.8%). The difference in the means between the control plants and regenerants was not statistically significant. We also observed that the duration of in vitro culture may affect the final recombinant protein production; however, even after four months of growing in vitro, regenerants can produce GFP at the levels of 6–15% TSP (Figure 8). Additionally, SDS–PAGE analysis with the protein extracts obtained from GFP-expressing foliage of the regenerants was carried out to confirm the fluorometric data (Figure 9). GFP was detected as the dominant protein band, and the RuBisCO (a large subunit) protein band was significantly reduced. An especially pronounced band was detected in the R1 regenerant, supporting fluorometric data.

The mean of recombinant GFP per fresh leaf weight was 0.541 ± 0.165 mg/g, and the highest value was 2 mg/g (Figure S5). As regenerant R1 data significantly differed from others, the mean was also calculated excluding R1 data, and it was 0.380 ± 0.037 mg GFP/g of fresh leaf weight (the variation range was 0.196–0.602 mg/g). Additionally, afterward, a total amount of the recombinant protein that can be harvested from one *N. benthamiana* regenerant was estimated. The average potential of the proposed system was 1.43 ± 0.91 mg GFP per plant (with consideration of R1 regenerant data), and 0.53 ± 0.08 mg GFP per plant (variation range was 0.248–0.909 mg/plant) if R1 data were excluded from the calculations. However, in our best results, about 9.5 mg of GFP could be obtained from the foliage of one regenerant (Figure S6).

## 3. Discussion

In this study, a new simple approach was proposed to create plant tissue culture and many plants transiently expressing the target reporter protein GFP at high levels for a long time. A proposed system based on the long-distance systemic distribution of potato virus X RNA with the target gene in plant tissues. This system combines the advantages of transient expression technology and plant cell cultures. Nowadays, micropropagation is a routine procedure made by skilled technicians, and the methods for obtaining tissue cultures and the plant regeneration protocols are well developed for different plant species [39,40], including *Nicotiana* spp. [41,42,43,44,45]. Here, a common MS nutrient medium [38] supplemented with a simple combination of the plant growth regulators, such as naphthaleneacetic acid and benzylaminopurine, was used for effective in vitro regeneration of *N. benthamiana* plants [37]. Plants growing in aseptic in vitro conditions are maintained in a more stable state than those that grow in greenhouses: (i) growth medium composition is precisely described; thus, nutrient content variations are eliminated and (ii) plants are free of exogenous pathogens, such as insects, bacteria, or fungi, if some preventive methods are carefully carried out [46]. Pathogen attack that occurs in greenhouses can limit the recombinant protein production, as plant resources switch to pathogen defense mechanisms. Maintaining the plants producing the recombinant proteins under well-controlled in vitro conditions is preferable, especially when the final product must be standardized for pharmaceutical purposes, according to good manufacturing practices (GMPs). When in vitro tissue culture has been established, it is possible to increase the number of plants via micropropagation, or aseptic plants can be placed on the regeneration medium again to obtain the new “rejuvenated” GFP-expressing plants. Both methods were successfully tested in this study. Moreover, it is possible to establish a collection of in vitro plants producing different therapeutic proteins and to propagate them when needed, for instance, before the seasonal diseases. It is well known that somaclonal variations can occur in long-term tissue cultures and the regenerants obtained through callus tissues are prone to epigenetic changes (mutations) [47]; however, in our case, the variations occur since the beginning of the infection process, as not all cells are infected by viral RNA on the same leaf (explant). The obtained regenerants showed variability in GFP expression levels, but the variability in GFP expression levels was observed also after agroinfiltration of plants grown from the seeds. On the other hand, somaclonal variations of tissue cultures can have some advantages and can help to select and multiply the regenerants with the best characteristics, e.g., recombinant protein productivity and growth rate.

Our new system can be an alternative approach to widely used *Agrobacterium-*mediated transient gene expression. Agroinfiltration is the main approach used for the insertion of expression vectors in the plants to produce the recombinant proteins via transient gene expression: infiltration with a needleless syringe is a common method for laboratory studies [4,7,8,16,29,48], whereas vacuum infiltration is used for large commercial facilities (Medicago, Quebec, Canada; PlantForm, Toronto, Canada; Icon Genetics GmbH, Halle, Germany; Nomad Bioscience GmbH, Halle, Germany; [8]). Regardless, all plants must be agroinfiltrated. A new method eliminates the need to infect the plants regularly: several agroinfiltrated plants (needed for an initial step of plant tissue culture establishing) are enough for obtaining many agrobacteria-free regenerants producing the target protein in vitro. The regenerants growing in vitro can be successfully transferred in soil, restore adult plant phenotype, and produce high levels of the recombinant protein. The described method can be safer for nature, as it alleviates the necessity to use a thousand liters of agrobacterial suspension required for large commercial capacities. Here, a PVX-based vector was incorporated into agrobacterial cells to facilitate an initial infection procedure, but in principle, this system can work with direct mechanical rubbing of viral RNA transcripts.

Post-transcriptional gene silencing (PTGS) is a common mechanism of plant defense against viruses [49], transposable elements, and transgenes [50]. A mobile signal triggering RNA silencing can be transmitted over long distances via plasmodesmatal and phloem channels [51]; thus, transgene silencing occurs both in the transgenic plants [52], and in the plants infected with vectors for transient gene expression [53,54]. It was reported that some proteins that originated from the different pathogenic phytoviruses demonstrate anti-PTGS activity. One of the well-known suppressors of gene silencing is the p25 protein of PVX [55]. In this study, the *p25* gene was included in PVX-based vector construction; therefore, movement of viral RNA transcript and suppression of PTGS was provided by p25 protein. Earlier, a group of scientists tried to obtain transgenic tobacco plants with high expression levels of β-glucuronidase protein using similar PVX-based expression cassettes, but their attempt failed because of PTGS, which was especially pronounced in the lines with the mutated *p25* gene [33]. Our results suggest that the PTGS mechanism works slower when plants are grown in aseptic conditions in vitro: the regenerants demonstrated quite stable GFP expression in leaves and roots within four to five months. In contrast, when the regenerants were transferred to soil, PTGS occurred faster, and a notable decrease in green fluorescence was detected after 6–8 weeks. A molecular mechanism underlying the delay of PTGS for the in vitro growing regenerants remains unclear: nutrient medium chemicals [56,57] or plant physiological and developmental status may contribute to this process [58,59]. However, our data contradict the hypothesis that PTGS invokes by highly expressed transgenes [58], as in this work, the GFP accumulation in the regenerants growing in vitro reached high levels. Moreover, another paper described a similar in vitro approach, but the transgenic tobacco plants with PVX “amplicons” were used instead of classical transient expression, and the researchers did not achieve significant transgene expression because of the high level of PTGS in all tested lines [60].

It was observed that the regenerants growing in vitro showed delaying in the growth rate. In contrast, most regenerants restored their adult plant phenotype after 4–6 weeks of growing in soil. The regenerants with normal adult phenotype can increase the total amount of recombinant proteins produced per plant. Surprisingly, but the regenerants had different phenotypes (normal or dwarf) after growing in soil for a month that did not correlate with the recombinant protein yield. Phytohormones are known to play an important role in plant growth, while it was shown that exogenous auxins and cytokinin do not significantly influence transient gene expression [61]. Irreversible dwarfism can occur because of the plant response to the pathogen attack (viral RNA transcripts) that leads to changes in the phytohormone balance, e.g., via interfering with phytohormone biosynthesis, by altering or blocking important components of the phytohormone signaling pathways, as it was shown for the fungi pathogens [62,63,64], or because of changes in the regulation of the host gene expression levels [65].

Earlier, it was reported that the recombinant protein production through transient gene expression depends on leaf position: the highest levels were detected in the upper leaves [66,67], specifically from the first to sixth leaves from the apex [3]. We observed similar results in control agroinfiltrated plants and experimental regenerated plants: the best GFP production was detected in the upper leaves started from the “zeroeth” leaf (not fully expanded upper leaf) to the fourth leaf from the apex. These results were not surprising, as they reflect the features typical for leaf development: young leaves expand rapidly, undergoing fast protein synthesis, whereas in mature leaves, protein metabolism is at a consistently low level, and in senescent leaves proteins are intensively degraded [68].

Many studies have shown that plant expression vectors, based on the backbones of plant self-replicating viruses, lead to high yields of recombinant protein, including proteins favorable for medicine uses [4,12,13,14,15,16,19,69]. As in this study, a PVX-based vector was used, it was not surprising that the high levels of reporter green fluorescent protein were detected in the individual upper leaves (up to 70–80% of the total soluble proteins). The same high levels of GFP (up to 70–80% or up to 3.7–5 mg/g of fresh leaf weight) were reported for plant expression vectors based on the other virus backbones [2,3,70]. While the upper leaves synthesize more protein, unfortunately, they are usually smaller than lower ones; thus, the final target protein production obtained from the whole plant will be diminished. To be more accurate in our calculations, we estimated the recombinant protein yield obtained from all GFP-expressing foliage of a regenerant. The best result of GFP production reached up to 47% of the total soluble proteins, indicating the good potential of the proposed method for obtaining other recombinant proteins after some optimization of the protocol. Our further experiments will include testing the described system for the production of other recombinant proteins: we plan to obtain tissue culture and to determine the content of recombinant protein and its toxicity to the plant cells or whole plants growing in vitro.

A possible future improvement includes the selection of the best-producing regenerants and their subsequent micropropagation. On the other hand, greenhouse conditions can influence protein production as well, so further increasing the recombinant protein levels in greenhouses is possible via optimization of physical parameters (light intensity or spectrum, temperature, humidity) [66,71,72]. As our system includes a PVX-based vector, the physical factors affecting the PVX infection process may also affect target gene expression and recombinant protein synthesis. Our preliminary results showed that bright GFP fluorescence almost completely disappeared in the regenerants growing at 30 °C during a week, but it recovered in the upper young leaves when the temperature was decreased to 25 °C. These results may reflect the relationship between the temperature and the PVX infection process: systemic infection was observed at 15–25 °C, and no PVX was detected in plants growing at 30 °C [73]. Another study showed the correlation between the light intensity and the PVX infection in potato plants [74]; thus, this factor may also affect recombinant protein yield.

In this manuscript, an average time required for obtaining the recombinant proteins in *N. benthamiana* plants via in vitro tissue culture was estimated (summarized in Table 1). Briefly, the maximum time (from the initial step of plants growing from seeds) is six to seven months, and the minimum time (when tissue culture is established) is two months (from the regeneration step) or one month (when plants with roots grow in vitro). In comparison, for obtaining the recombinant proteins via widely used *Agrobacterium-*mediated transient gene expression, one and a half months is needed for growing *N. benthamiana* plants before agroinfiltration and then one to two weeks for protein accumulation after agroinfiltration. In total, about two months are needed to obtain recombinant proteins via *Agrobacterium-*mediated transient gene expression as well. The total time required for obtaining tissue culture producing recombinant protein through transient expression is almost the same as the time needed for obtaining transgenic plants, and with a transient expression, we cannot obtain productive seeds. However, the transient gene expression typically shows much higher levels: the best results demonstrated for the transplastomic plants were about 30% TSP [75] and were lower for the nucleus transformants; in contrast, transient gene expression can reach up to 50–80%TSP ([2,3,4], our data).

## 4. Conclusions

The summarized advantages of the proposed in vitro system are as follows: (i) long-term recombinant protein production due to delayed gene silencing; (ii) high levels of recombinant protein obtained through transient expression; (iii) regenerated plants are free from agrobacteria; (iv) recombinant proteins produce under well-controlled conditions; (v) regenerants can be transferred to soil for increasing total recombinant protein yield. The system can be a new technique for plant molecular farming, combining the advantages of transient expression and cell tissue cultures.

## 5. Materials and Methods

### 5.1. Genetic Constructions and Bacterial Strains

**Figure 10:**
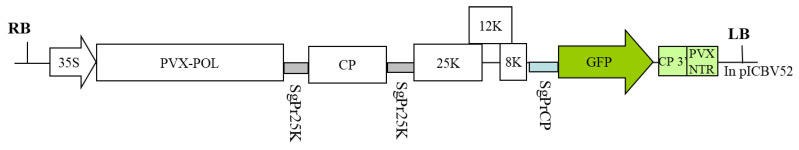
The graphic presentation of pICH27566 plasmid T-DNA: 35S—35S promoter of CaMV; *pvx-pol*—gene coding PVX RNA-directed RNA polymerase; SgPr25K — subgenomic promoter of PVX 25K protein gene; *cp*—gene coding PVX coat protein; *25k, 12k, 8k*—the triple gene block (genes coding PVX 25K, 12K, and 8K proteins); SgPrCP—subgenomic promoter of PVX coat protein gene; *gfp*—the *gfp* gene; CP 3’—3’ end of PVX coat protein; PVX NTR—3’UTR of PVX.

**Figure 11:**
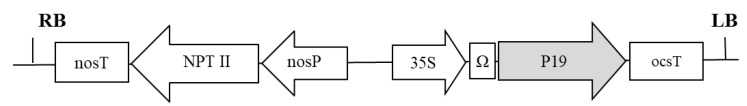
The graphic presentation of pICH6692 plasmid T-DNA: 35S—35S promoter of CaMV; Ω—5’omega untranslated region (translational enhancer); *p19*—gene of P19 protein (from tomato bushy stunt virus, suppressor of PTGS); ocsT—terminator of octopine synthase gene (from *A. tumefaciens*); nosP, nosT—promoter and terminator of nopaline synthase gene (from *A. tumefaciens*); *npt II*—gene coding neomycin phosphotransferase II.

All plasmid vectors used in this work were generously donated for scientific purposes by Nomad Bioscience GmbH Company (Halle, Germany). The PVX-based plasmid pICH27566 contained the potexviral RNA-directed RNA polymerase gene driven by 35S promoter of cauliflower mosaic virus (CaMV), followed by the coat protein (CP) gene and the triple gene block driven by *p25* subgenomic promoters, and the *gfp* gene, coding green fluorescent protein (GFP), driven by viral CP subgenomic promoter (for graphic vector details see Figure 10 and [76]). The plasmid pICH6692 contained the *p19* gene, coding a suppressor of post-transcriptional gene silencing from tomato bushy stunt virus, driven by 35S promoter of CaMV (Figure 11 and [77]). All vector constructions were transformed into *Agrobacterium tumefaciens* strain GV3101.

### 5.2. Plant Material and Growth Conditions in Greenhouse

*N. benthamiana* and *N. excelsior* seeds were obtained from the National Germplasm Bank of World Flora of the Institute of Cell Biology and Genetic Engineering (Kyiv, Ukraine). Seeds were germinated in commercial soil, and plants were grown in greenhouse conditions, i.e., 16 h light photoperiod at 24–26 °C, 3000–4000 lux. Six-to-eight-week-old plants were used for the infiltration procedure. The regenerated plants transferred from in vitro to soil were grown at the same greenhouse conditions.

### 5.3. Agroinfiltration Procedure

Preparation of agrobacteria for infiltration was made as described in [3]. Briefly, bacterial suspensions were grown overnight on an orbital shaker at 28 °C, precipitated by centrifugation at 6000 g, and resuspended in the infiltration buffer (10 mM MES, pH = 5.6, 10 mM MgSO_4_, 100 µM acetosyringone) to OD600 = 1.0. Agrobacteria harboring plasmids with *gfp* and *p19* genes were mixed in equal volumes (pICH27566 + pICH6692), as controls were used buffer only or agrobacterial cells carrying a plasmid with *p19* gene (pICH6692) only. Leaves were agroinfiltrated using a needleless syringe.

### 5.4. In Vitro Tissue Culture Establishment, Plant Regeneration, and Micropropagation

When green fluorescence was visible in all areas of the upper systemic young leaves, leaves were cut off, washed with a washing soap, and rinsed thoroughly 2–3 min in running water, repeated two-three times. Then, leaves were transferred in a laminar flow cabinet, and the following procedures were carried out under sterile conditions: sterilized in 70% ethanol about 60 s, two times rinsed thoroughly in sterile distilled water, cut to two to three pieces (explants), dried on a sterile filter paper for a few minutes, and put on the regeneration medium (MSR). MSR was the nutrition agar Murashige and Skoog (MS) medium [38] supplemented with 0.1 mg/L of 1-Naphthaleneacetic acid (Merck, Kenilworth, NJ, USA) and 1 mg/L of 6-Benzylaminopurine (Sigma-Aldrich, St. Louis, MO, USA) [36,37], and with 700 mg/L cefotaxime (or without antibiotic for tissue culture “rejuvenation”). Petry dishes with the explants were placed in a growth chamber at 25–26 °C and 16 h light photoperiod. The obtained regenerants were maintained in vitro on MS medium without any supplements. The regenerants with roots were transferred from in vitro to soil, adapted to the greenhouse conditions (2–3 days), and then grew normally for 1–1.5 months. In total, 10 regenerants of different ages (2–5.5 months old) were used for the greenhouse experiments to assess recombinant protein production.

### 5.5. DNA Isolation and PCR Analysis

For PCR analysis, genomic DNA from the plant material was isolated as described in [78]. The manufacturer’s guidelines were used for PCR mix preparation and for thermal cycling conditions (product DreamTaq DNA Polymerase, Thermo Scientific, Waltham, MA, USA).

Primers used in this work:

Actin F1: ctggagatgatgctccaag; Actin R1: gatagaacggcttgaatgg; expected fragment size was 352 bp (the housekeeping *actin* gene detection—the control of total DNA isolation).

VirD1-1 (F): atgtcgcaaggcagtaagccca; VirD1-2 (R): ggagtctttcagcatggagcaa; expected fragment size was 438 bp (for the agrobacterial *vir D1* gene detection—the control of agrobacterial contamination).

GFP For4 (F): atggtgagcaagggcgag; GFP Rev2 (R): ccatgccgtgagtgatcc; expected fragment size was 703 bp (for the *gfp* gene detection—the control to confirm transient transformation and failure of *gfp* gene integration in plant genome).

### 5.6. GFP Detection, Isolation, and Analysis

*Detection and measurement of GFP content.* The presence of GFP in the leaves was visually detected with a hand-held black ray lamp (UVP, Upland, CA, USA) or UV diodes as green fluorescence on the dark red background. Photos were made by SAMSUNG NX1100 camera, using an orange filter for UV light photos if otherwise not specified. Fluorescence in the attached leaves of the regenerants was measured on a spectrofluorometer (Fluorat-02, PANORAMA, Lumex-Marketing, Saint Petersburg, Russia) via an additional device for non-cuvette measurements. For this measurement, 3–5 dot replicates were made on the same leaf, and then an average mean was used. Fluorescence in the protein extracts was measured on the same spectrofluorometer, and data were calculated according to the standard calibration curve based on purified GFP. The excitation peak of GFP was at 395 nm, and the emission peak was at 510 nm.

*Extraction of the total soluble proteins from fresh leaves*. The experimental and control leaves were cut off, weighted, and ground in a prechilled mortal with a pestle on ice or in a laboratory mixer mill in prechilled metal tubes with balls (type MM 400, RETSCH, Haan, Germany, vibration frequency was 25 Hz for 5 min) in two volumes (w/v) of ice-cold extraction buffer: 100 mM Tris/HCl, pH 8.0, 5 mM Na_2_EDTA, 100 mM NaCl, 2.5% polyvinylpyrrolidone [18] plus 250 mM sucrose. Debris was precipitated by two rounds of centrifugation at 13,000 g for 10 min, at 4 °C. The clear supernatant was used for the further assays. The total soluble proteins in the extracts were calculated according to the Bradford method, using bovine serum albumin as a standard [79].

*Protein electrophoresis***.** The native and reducing SDS–PAGE analyses of the crude leaf extracts contained recombinant protein and controls were carried out as described in [3].

Statistical data were calculated in the Excel 2016 program (Microsoft office software). Data were presented as the mean ± standard error. Student’s *t*-test was used to compare the difference in control and experimental groups. From 6 to 17 replications were made for the statistical data calculations.

## Figures and Tables

**Table 1:** Stages and time required for obtaining the recombinant proteins in *Nicotiana benthamiana* regenerants through in vitro tissue culture.

Stage	Time Required (Days)
Plant growing prior to infection	35–55
An agroinfiltration procedure	1–2
System infection and transient gene expression in the upper leaves	5–10
Transferring leaves to in vitro conditions	1
Shoots regeneration	14–45
Roots appearing	14–30
Cycle step to “rejuvenate” plant tissue culture: leaves of regenerants growing in vitro can be put on a regeneration medium to obtain the young regenerants.	
It is possible to postpone the next step and maintain the regenerants in vitro for up to 6 months.	
Transferring the regenerants to soil and yielding the recombinant protein from the whole plant	35–50
Total time required to obtain the recombinant protein	Minimum—2 months (when tissue culture is established)Maximum—6–7 months (starting from the plant growing stage)

## Data Availability

The data are available within the article or its Supplementary Materials.

## Supplementary Materials

The following are available online at https://www.mdpi.com/article/10.3390/plants10102187/s1, Figure S1: PCR analysis for the presence of the *virD1* gene and the *actin* gene, Figure S2: PCR analysis for the presence of the target *gfp* gene and the *actin* gene, Figure S3: Regenerant R1 grown in a greenhouse for a month under visible and UV light, Figure S4: Fluorometric measurement data of protein extracts prepared from the total GFP-expressing foliage of regenerants with 1/50 extract dilution, Figure S5: GFP content in *N. benthamiana* regenerants calculated as recombinant protein amount per fresh leaf weight, Figure S6: GFP content in *N. benthamiana* regenerants calculated as recombinant protein amount collected from total GFP-expressing foliage of plant.
