# Supplementary material for: Long-Term Potato Virus X (PVX)-Based Transient Expression of Recombinant GFP Protein in Nicotiana benthamiana Culture In Vitro
Source: Plants (Basel). 2021 Oct 15;10(10):2187. doi: 10.3390/plants10102187 (PMC8537016; doi:10.3390/plants10102187)
Supplement: Supplementary file 1 [file plants-10-02187-s001.zip › plants-1393474-supplementary.pdf]

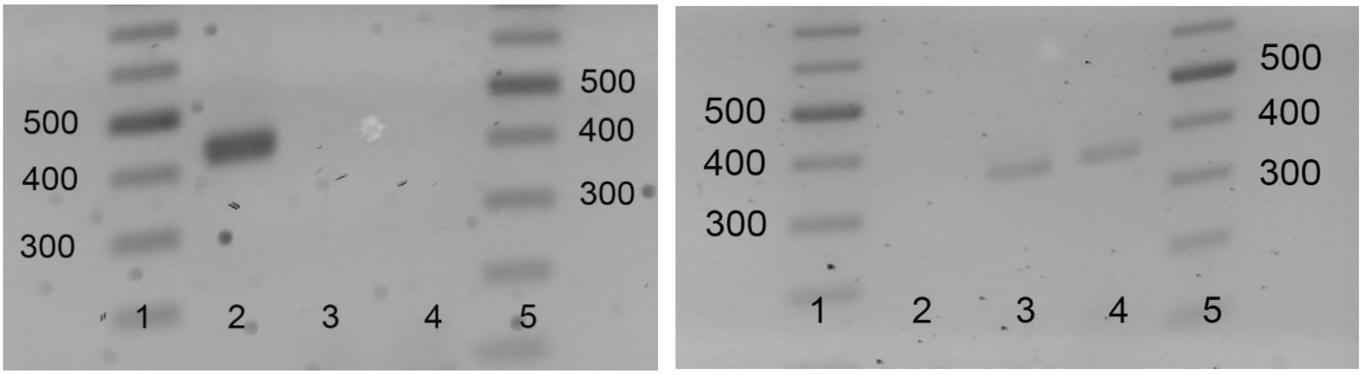


**Figure S1.** PCR analysis for the presence of the *virD1* gene (left) and the *actin* gene (right): 1,5—100 bp DNA ladder, size in bp; 2—agrobacterial DNA (control); 3,4—DNA samples isolated from GFP-expressing regenerants. The *virD1* gene expected size is 438 bp (control of agrobacterial contamination); the *actin* gene expected size is 352 bp (housekeeping gene).

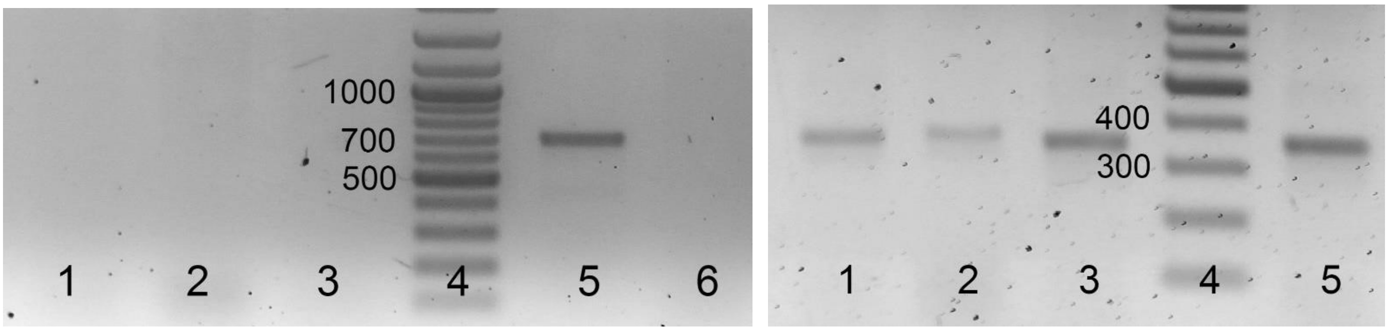


(**a**) (**b**)

**Figure S2.** PCR analysis for the presence of the target *gfp* gene (**a**) and the *actin* gene (**b**): 1–3—the DNA samples isolated from GFP-expressing regenerants; 4 —100 bp DNA ladder, size in bp; 6**a** and 5**b**—the DNA sample isolated from the negative control wild-type plant (without any infection); 5**a**—positive control for the *gfp* gene (pICH27566 plasmid used in this work). The *gfp* gene expected size is 703 bp (the control of transgene integration in the plant genome); the *actin* gene expected size is 352 bp (the housekeeping gene).

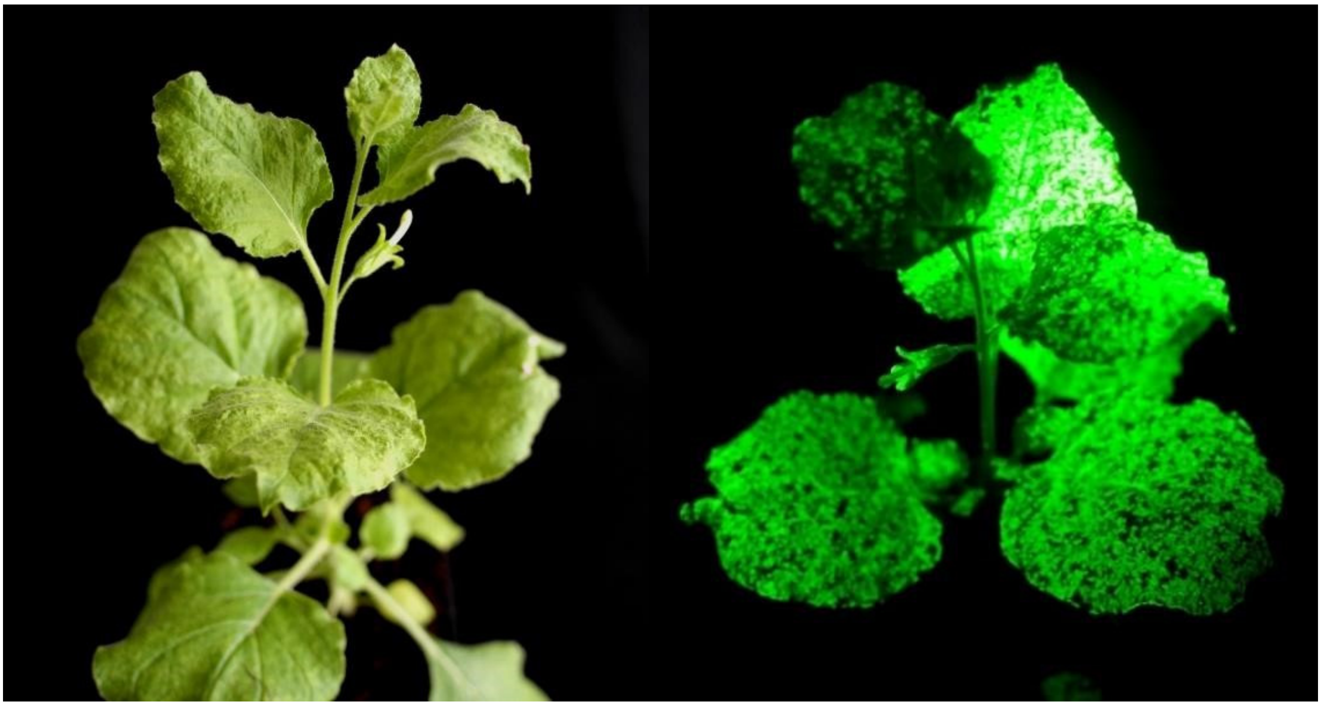

**Figure S3.** Regenerant R1 grown in a greenhouse for a month under visible (left) and UV (right) light. Green fluorescence (under UV light) indicates GFP accumulation. Photo in UV light was made using an orange filter.

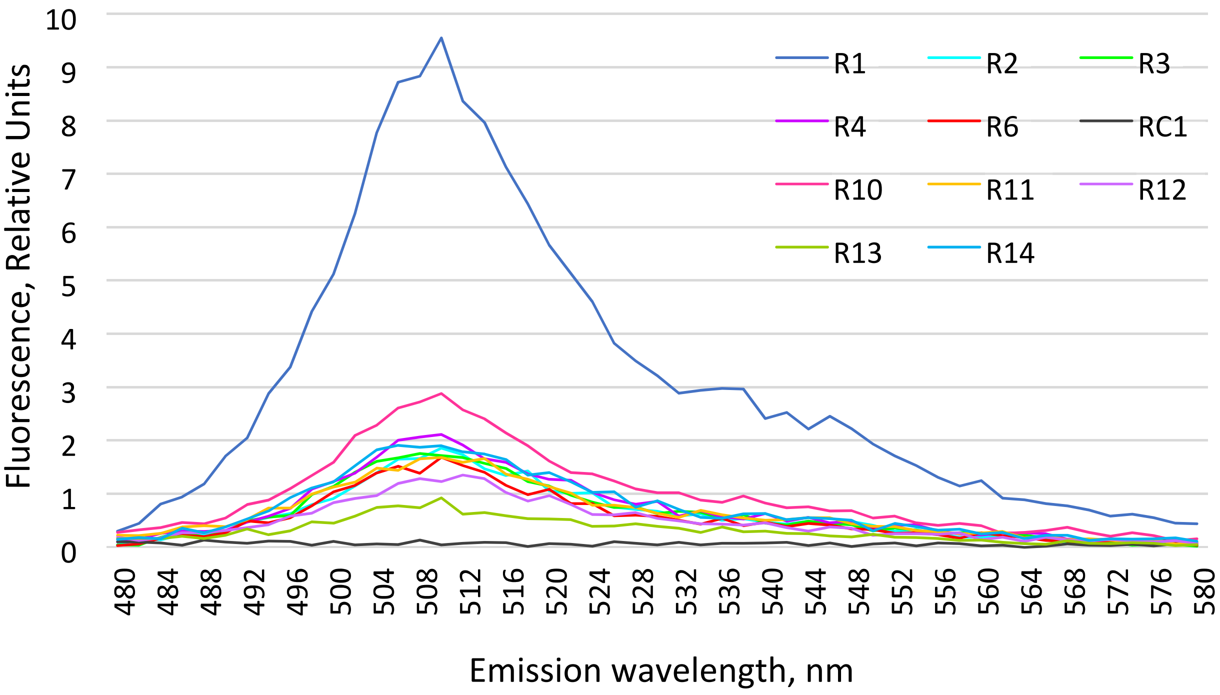


**Figure S4.** Fluorometric measurement data of protein extracts prepared from the total GFP-expressing foliage of regenerants with 1/50 extract dilution: R1–R14—tested regenerants grown in the greenhouse; RC1—negative control regenerant without viral RNA. Measurements were made at an excitation wavelength of 395 nm, and the GFP emission peak is 510 nm.

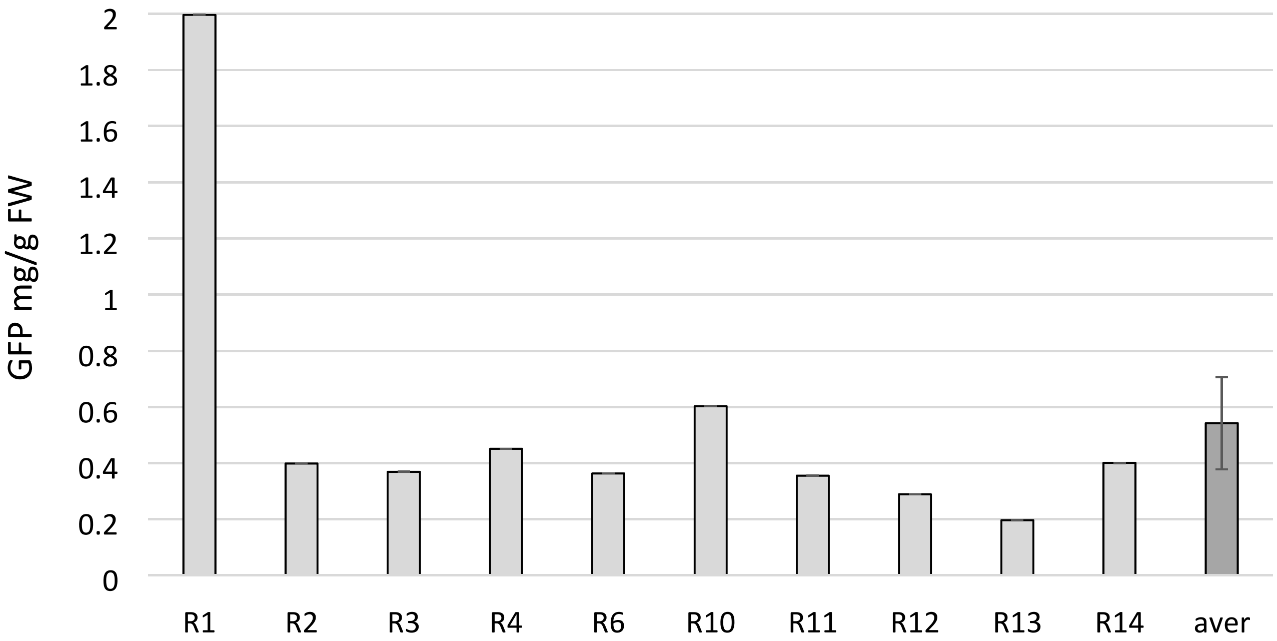


**Figure S5.** GFP content in *N. benthamiana* regenerants calculated as recombinant protein amount per fresh leaf weight (FW): R1–R14—tested regenerants grown 1–1.5 months in the greenhouse, arev – average mean. Bar means SE.

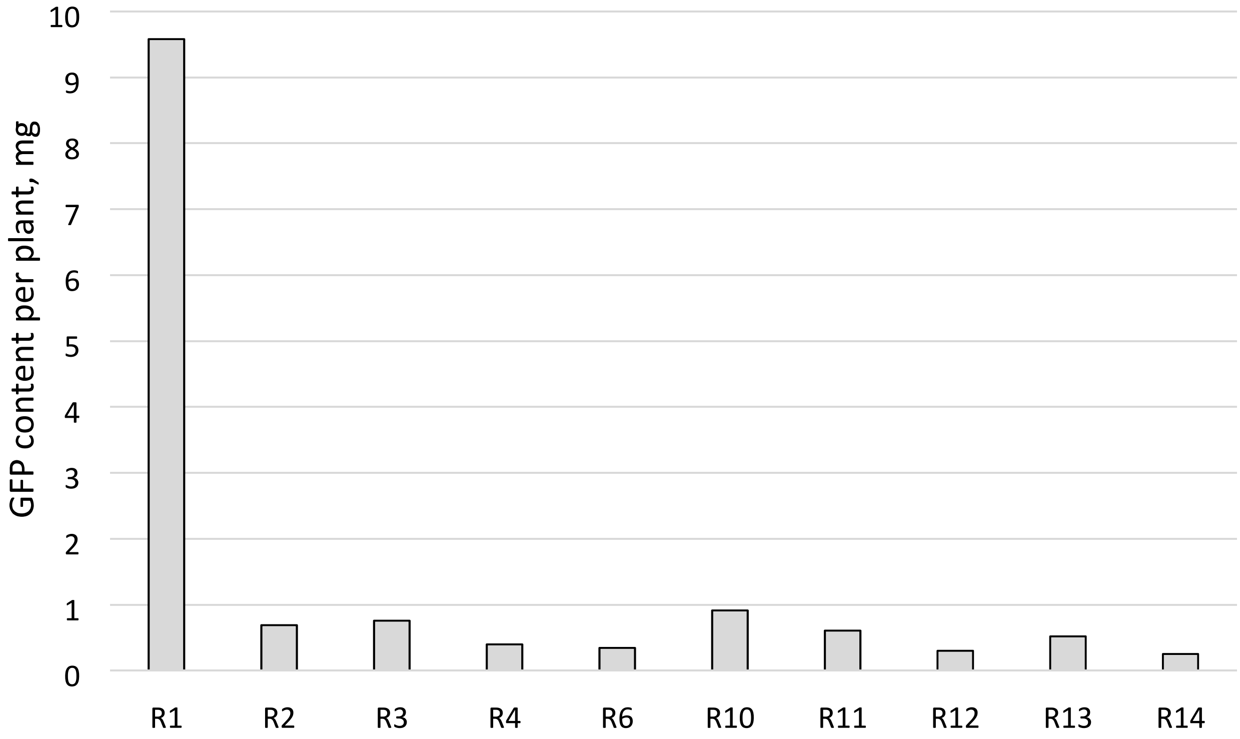


**Figure S6.** GFP content in *N. benthamiana* regenerants calculated as recombinant protein amount collected from total GFP-expressing foliage of plant: R1–R14—tested regenerants grown 1–1.5 months in the greenhouse.
